# Considerations for Low Phospholipid-Associated Cholelithiasis (LPAC) Syndrome: Report of Three Cases

**DOI:** 10.7759/cureus.48082

**Published:** 2023-10-31

**Authors:** Najoua El Moutaoukil, Mehdi Zouaoui, Youssef Hnach, Mbarek Azouaoui, Nourdin Aqodad

**Affiliations:** 1 Gastroenterology and Hepatology, University Hospital Souss Massa, University Ibn Zohr, Faculty of Medicine and Pharmacy, Agadir, MAR

**Keywords:** case report, ursodeoxycholic acid, comet tail, genetic, cholelithiaisis

## Abstract

Low phospholipid-associated cholelithiasis (LPAC) syndrome is a rare underdiagnosed genetic feature presenting less than 1% of symptomatic cholelithiasis, with variable clinical forms ranging from simple to complications. Diagnosis criteria are recurrent biliary symptomatology occurring in young patients (<40 years old) and/or recurrence after cholecystectomy and/or having a history of biliary gallstones in first-degree relatives and characteristic ultrasound findings. Early detection of this entity, due to the awareness of gastroenterologists, radiologists, and visceral surgeons, will allow an improvement in the quality of life of patients and the prevention of complications. We report three cases of the LPAC syndrome and discuss its different aspects.

## Introduction

Low phospholipid-associated cholelithiasis (LPAC) syndrome is a genetic disease characterized by a decrease in the content of phospholipids in the bile, leading to a defect in the solubilization of cholesterol, resulting in the formation of both vesicular and intrahepatic cholesterol lithiasis [1].

It is a particular form of cholelithiasis in young, non-overweight subjects, described in 2001 by the team of Saint-Antoine Hospital in Paris [2]. Its prevalence is estimated at 1% in symptomatic cholelithiasis patients, according to a last retrospective cohort published in 2021 [3]. We report and analyze through three different case reports and a literature review the clinical, radiological, and evolutionary aspects of this entity.

## Case presentation

Case 1

**Figure 1:**
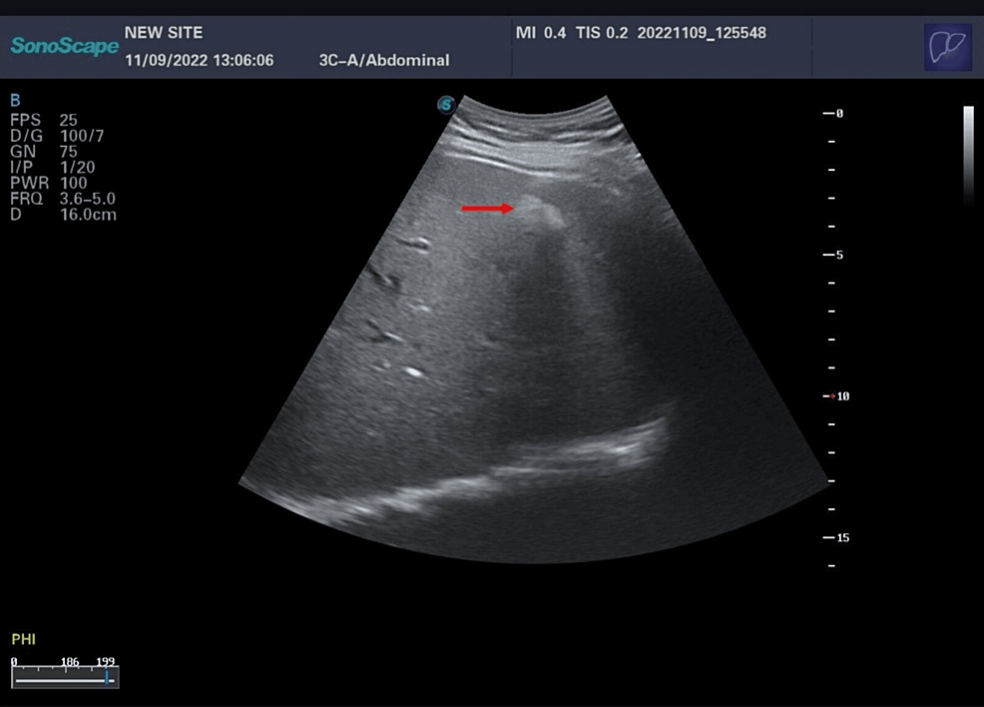
Intrahepatic macrocalculi with posterior shadow cone (red arrow)

**Figure 2:**
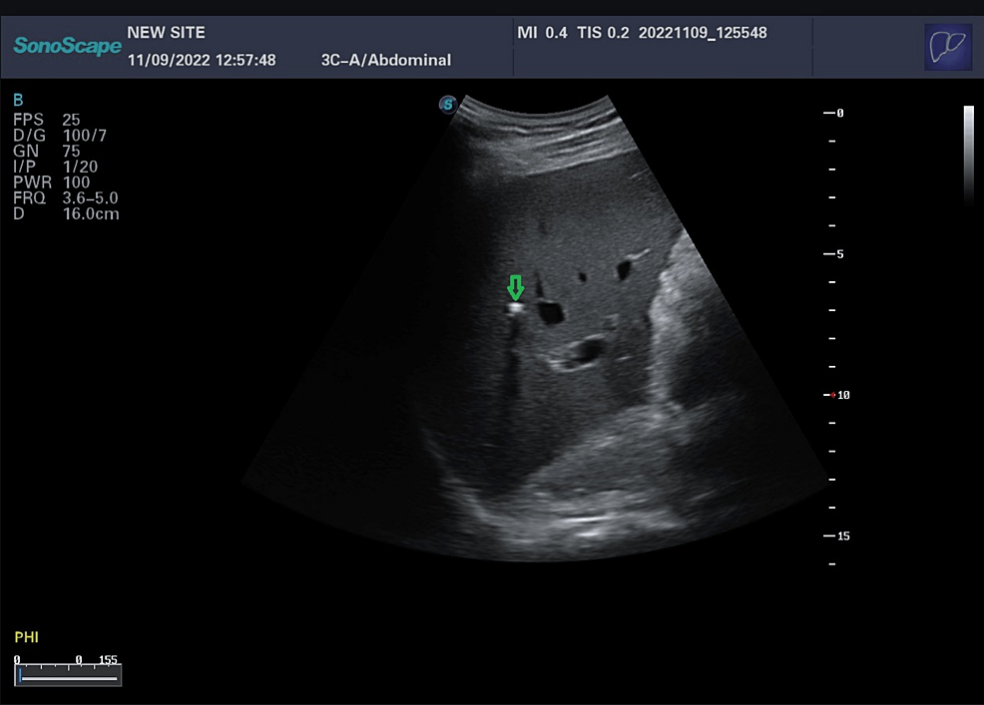
Intrahepatic microcalculi with posterior shadow cone (green arrow)

We report the case of a 40-year-old man, a chronic active smoker with 10 pack-years, nonalcoholic, complaining about recurrent biliary colic without a family history of cholelithiasis or neoplasia. He was admitted to the emergency department with severe acute pancreatitis and an initial systemic inflammatory response syndrome. His Bedside Index of Severity in Acute Pancreatitis (BISAP) score was 2, and his body mass index (BMI) was 23.6 kg/m². Physical examination was normal except for epigastric tenderness. Biological assessment on admission (24 hours after the pain) showed a blood lipase level of 30 x ULN (upper limit of normal), no cytolysis, slightly elevated gamma-glutamyl transferase (GGT) of 1.4 x ULN, and normal triglyceride and calcium levels. Abdominal ultrasound (US) revealed images of intrahepatic micro- and macro-stones with posterior acoustic shadow (Figures 1, 2) and a thin-walled gallbladder (GB) with sludge, without dilatation of intra- or extra-hepatic bile ducts and a heterogeneous pancreas.

**Figure 3:**
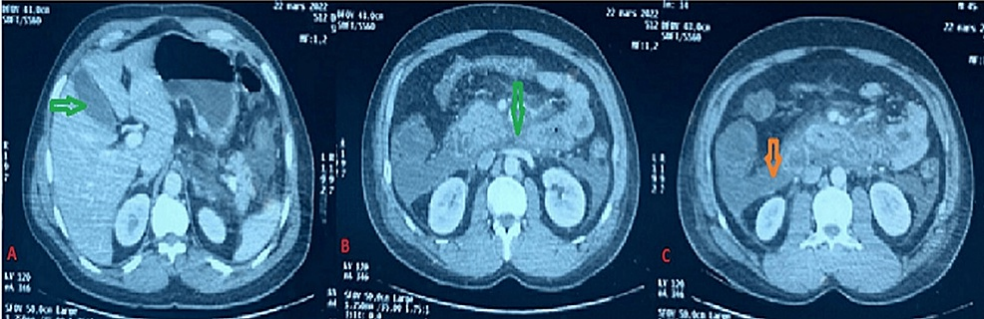
Abdominal and pelvic CT at 96 hours of pain A: Gallbladder containing sludge (green arrow) without evidence of intrahepatic stones, B: intrapancreatic necrosis (green arrow), C: perirenal fat necrosis secondary to biliary pancreatitis (orange arrow)

Abdominal and pelvic CT at 96 hours of pain concluded that the CT severity index score (CTSI) was 10 and that GB contained sludge, without evidence of intrahepatic stones (Figure 3).

In view of the young age of the patient, the absence of risk factors for cholelithiasis, recurrent biliary symptoms, and the characteristic US images, the diagnosis of LPAC syndrome was made, and the patient was managed by UDCA (ursodeoxycholic acid) 1000 mg/d for the long term without cholecystectomy. After 3 months of treatment, clinical improvement was noted and GGT was normal at 13 IU/l.

Case 2

**Figure 4:**
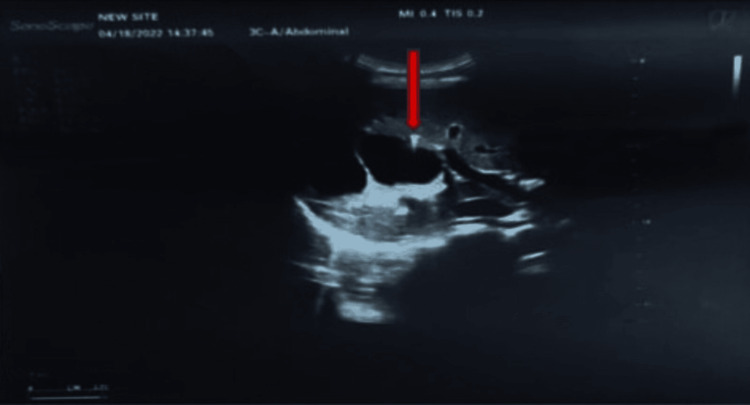
Abdominal ultrasound Hyperechoic foci, with comet tail artifacts (red arrow) and hydatid cyst between segments VI and VII measuring 80.5 x 68 x 60 mm, classified as CE2 according to WHO classification

A 16-year-old patient, appendectomized 1 year ago, with a history of cholelithiasis in his mother, brother, and sister at a young age, presented with recurrent biliary pain. Physical examination was normal and his BMI was 16 kg/m². The blood count was normal except for hypereosinophilia of 720/mm3. There was no cytolysis nor cholestasis and the lipid profile was normal. Abdominal US showed gallstones, without either intrahepatic or main bile duct (MBD) dilatation. We noted the presence of hyperechoic foci, with comet tail artifacts in the intrahepatic area (Figure 4).

The US also showed a hydatid cyst between segments VI and VII measuring 80.5 x 68 x 60 mm, classified as CE2 according to the WHO classification, and the hydatid serologic test was positive.

Given the young age of our patient, typical US images, and cholelithiasis in first-degree relatives, the diagnosis of LPAC syndrome was made, and then he was managed by UDCA 10 mg/d over the long term with favorable outcomes.

Case 3

A 27-year-old female patient, G4P3, on 3 months postpartum, presented with recent biliary colic with clinical cholestasis. In addition, the patient reported the occurrence of unexplored pruritus during the third trimester of pregnancy, and premature delivery at 32 weeks of amenorrhea. Clinical examination revealed a BMI of 29.3 kg/m² and tenderness of the right hypochondrium. On biological assessment, there was cytolysis (aspartate aminotransferase (AST) 2 x ULN, alanine aminotransferase (ALT) 3.8 x ULN) and cholestasis (total bilirubin: 20 mg/l, direct bilirubin: 17 mg/l, GGT: 13 x ULN, alkaline phosphatase (ALP) 1.6 x ULN). Hepatitis B and C testing were negative, and protein electrophoresis and IgG4 were normal. Abdominal US showed multiple hyperechoic intrahepatic "comet-tail" spots compatible with LPAC syndrome, sludge in the MBD that was not dilated, and gallstones.

**Figure 5:**
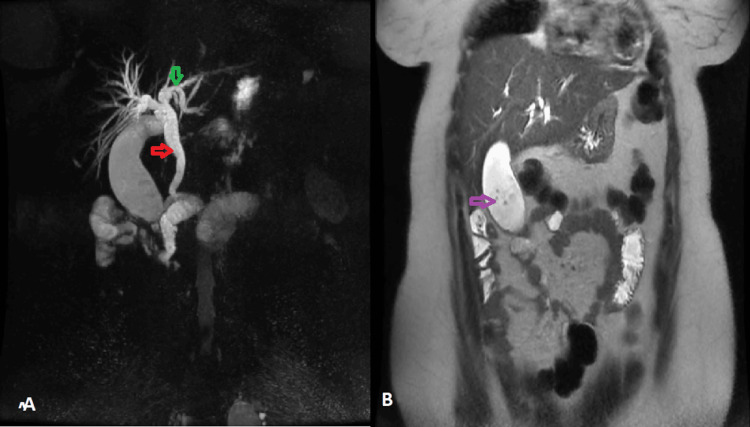
Magnetic resonance cholangiopancreatography A: 3D MRI showing dilated main bile duct at 9 mm (red arrow) and dilatation of intrahepatic bile ducts (green arrow), B: coronal slice in T2 showing gallstones (purple arrow) and absence of visualization of intrahepatic stones on MRI.

Magnetic resonance cholangiopancreatography showed adilated MBD at 9 mm with homogeneous contents, and edema of the papilla probably due to the oddities (Figure 5).

Because of the suspicion of LPAC syndrome, she was managed by UDCA at a progressive dose until reaching 10 mg/kg/d + trimebutine and cholestyramine, with a regular biological control. The clinical course was favorable. Liver biology at 1 month showed normal AST and ALT, GGT 1.6 x ULN, ALP 1.8 x ULN, and total bilirubin 14.

## Discussion

The three main components of bile - phospholipids, cholesterol, and bile acids - are secreted into the bile by transporters and these transporters allow cholesterol to dissolve in micelles. Mutation of the *ABCB4* gene (ATP-binding cassette subfamily B member 4), located on chromosome 7 locus 21 (7q21), leads to dysfunction of the MDR3 protein, a phospholipid transporter at the canalicular pole of hepatocytes. Therefore, the bile produced is low in phosphatidylcholine, the major phospholipid in human bile. As a consequence, bile exhibits a high degree of lithogenicity, containing a high concentration of cholesterol crystals that precipitate in bile ducts, resulting in the formation of intra- and extrahepatic cholesterol stones [4]. The bile secreted also has a strong detergent effect due to the excess of bile acids, inducing inflammation of the biliary epithelium and elevation of the GGT produced by cholangiocytes [5].

The *ABCB4* mutation is present in only 30% to 50% of cases, most often heterozygous, missense, and without correlation with the phenotype. Other mutations are probably involved but have not yet been detected. The spectrum of mutations in the *ABCB4 *gene is also responsible for gestational cholestasis and progressive familial intrahepatic cholestasis (PFIC 3) [6]. Due to the inhibitory effect of estrogen on MDR3 carriers, LPAC syndrome has a clear female presence. The average age at diagnosis is 29.1 years for women and 38.7 years for men [4]. The prevalence of LPAC syndrome is not well known; it is estimated, according to Dong et al., to be 1% in patients with symptomatic gallbladder lithiasis [3].

Clinical presentations can range from simple biliary pain to complications. The most frequent complication is lithiasis migration with elevation of transaminase levels (Case 3), which is not usually demonstrated by echo-endoscopy or MRI [1]. One patient out of five presented at least one episode of pancreatitis (Case 1). Acute cholangitis has been described in 25% of patients. Cholecystitis is not very rare and has been reported in 7% of cases [3].

In pregnancy, 50% of women have cholestasis during the second or third trimester. This is shown by pruritus and biological cholestasis (*ABCB4 *mutations are encountered in 15% of cases of gestational cholestasis). Premature delivery may complicate gestational cholestasis in 2/3 of cases. Treatment with UDCA during pregnancy reduces the risk of exacerbation and prematurity [1].

Diagnostic criteria for LPAC syndrome were established by the Saint-Antoine team in 2003 [7]. The presence of two of the following items confirms the LPAC syndrome: (1) onset of symptoms in adults less than 40 years of age; (2) recurrence of symptoms after cholecystectomy (after eliminating residual MBD lithiasis and opioid consumption); and (3) radiological signs of intrahepatic lithiasis

Diagnostic criteria were developed from studies including small samples, whose main goal was detecting *ABCB4 *mutations, which is not constant. These criteria may be missing in more than half of patients, and are also based on the recurrence of symptoms after cholecystectomy, which could be avoided if the diagnosis was made earlier [3].

Other criteria have been proposed by Dong et al., including MBD lithiasis, normal weight, and absence of cholecystitis [3]. Picon et al. added other factors: early onset of symptoms <30 years of age, history of gestational cholestasis, and first-degree family history of cholelithiasis before 30 years [8]. In our experience, we were based on the French recommendations, which propose to associate at least two or more elements of the following criteria to retain the diagnosis of LPAC [5]: biliary pain; icterus due to obstruction of the main bile duct, or acute cholangitis or pancreatitis occurring before age 40; recurrence of these events after cholecystectomy; the presence of intrahepatic echogenic foci on US; history of gestational cholestasis; or family history of cholelithiasis in first-degree relatives.

Biological findings are not specific: chronic cholestasis can be found, particularly an elevation of GGT [1]. Genetic studies can be useful in situations of uncertainty, however, they are not essential for diagnostic confirmation due to their high expense and inaccessibility. Determination of phospholipids levels from bile collected by duodenal or choledochal aspiration is feasible, but it is only performed in specialized laboratories [1].

Ultrasound by an operator who has been trained to look for LPAC syndrome is the key examination to show intrahepatic calculi images. These images can be sludge or intrahepatic hyperechoic foci with either a posterior "comet tail" enhancement corresponding to ductal deposits of cholesterol crystals, or shadow cones corresponding to macro or micro stones. The "comet tail" image is the most characteristic - it must be distinguished from pneumobilia, which is mobile [9]. Color Doppler can reveal a "twinkling artifact", which is similar to the "comet tail" images in standard ultrasound [5].

Depending on the expertise level of the operator, the detection rate of radiological aspects of LPAC can range from 4% to 90%. Micro-lithiasis is often not found by CT or MRI; MRI can be helpful to rule out differential diagnoses, mainly Caroli disease (by the absence of the "central dot sign") and primary sclerosing cholangitis. The risk of secondary biliary cirrhosis and cholangiocarcinoma has been reported in the literature, but these complications are rare [4].

When clinical and imaging findings are characteristic, the diagnosis of LPAC is confirmed and treatment with UDCA should be initiated without further investigation. In cases of uncertainty, *ABCB4 *genotyping is recommended, but the absence of a mutation does not banish the diagnosis and a therapeutic test employing UDCA can be suggested [5].

UDCA is well tolerated and can be prescribed at a dose of 7 to 10 mg/kg/d. It could also be intensified to 20 mg/kg/d. In early forms, it reduces symptoms and improves them in advanced cases, but recurrence can occur when treatment is stopped. However, the disappearance of lithiasis is slower. The underlying hypothesis suggests that biliary symptoms are caused by inflammatory lesions of the cholangiolar epithelium and cholesterol microcalculi rather than macrocalculi [4]. The mechanism of action of UDCA is explained by the expression of the MDR3 transporter, enhancing the secretion of phosphatidylcholine and hydrophilic bile acids, which serve as protective agents for the cholangiolar membrane. Furthermore, it helps solubilize cholesterol in the bile and dissolve stones.

There is no strict diet recommended. In cases of associated hypercholesterolemia, statins are preferred over fibrates, which increase lithogenicity. Estrogen and progestin should be discontinued during the first weeks of treatment with UDCA because they inhibit the secretion of phospholipids and increase the symptoms [1]. Cholecystectomy should not be performed frequently in cases of pancreatitis, cholangitis, or lithiasis migration, except in cases of acute cholecystitis or persistent symptoms following UDCA [4]. An endoscopic approach is indicated in obstructive stones of MBD, or forms with multiple intrahepatic lithiases causing cholangitis or recurrent liver abscesses; biliary stents are placed in several hepatic sectors ensuring good biliary drainage or other lithotripsy maneuvers. If needed, a partial hepatectomy could be suggested in cases of abscess, massive hepatic lithiasis, recurrent angiocholitis, or neoplastic complications. Liver transplantation should be considered in decompensated biliary cirrhosis with jaundice and ascites [5].

Family screening is recommended for first-degree relatives over 18 years of age by US or by testing for the *ABCB4 *mutation (if detected in the index individual) to treat patients before symptoms and complications appear. A normal US scan in a young asymptomatic relative does not exclude the diagnosis and the scan must be repeated a few years later because radiological signs of LPAC syndrome may occur later [5].

## Conclusions

Through the presentation of our observations and a literature review, we have tried to make an overall assessment of LPAC syndrome, especially its clinical, morphological, and therapeutic aspects, to facilitate diagnosis and prevent severe complications.

We conclude that ultrasound performed by an experienced operator plays an essential role in the orientation of diagnosis, by displaying characteristic images. Therefore, an effective and simple treatment based on UDCA could be introduced and systematic cholecystectomy could be avoided.
